# Epidemiology and treatment outcomes in pediatric patients with post-injection paralysis

**DOI:** 10.1186/s12891-022-05664-4

**Published:** 2022-08-05

**Authors:** Siyou Song, Moses Fisha Muhumuza, Norgrove Penny, Coleen S. Sabatini

**Affiliations:** 1 University of California San Francisco, School of Medicine, San Francisco, CA USA; 2 Department of Orthopaedic Surgery, CoRSU Rehabilitation Hospital, Kisubi, Uganda; 3 University of British Columbia, Branch for Global Surgical Care, Vancouver, British Columbia Canada; 4 Department of Orthopaedic Surgery, University of California San Francisco, UCSF Benioff Children’s Hospital Oakland, 747 52nd Street, OPC 1st Floor, Oakland, CA 94609 USA; 5 UCSF Center for Health Equity in Surgery and Anesthesia (CHESA), San Francisco, California USA

**Keywords:** Post-injection paralysis, Intramuscular injection, Pediatrics, Iatrogenic disability

## Abstract

**Background:**

Post-injection paralysis (PIP) of the sciatic nerve is an iatrogenic paralysis that occurs after an intramuscular injection, with resultant foot deformity and disability. This study investigates the epidemiology and treatment of PIP in Uganda.

**Methods:**

Health records of pediatric patients surgically treated for PIP at the CoRSU Rehabilitation Hospital from 2013 to 2018 were retrospectively reviewed. Pre-operative demographics, perioperative management, and outcomes were coded and analyzed with descriptive statistics, chi-square for categorical variables, and linear models for continuous variables.

**Results:**

Four-hundred and two pediatric patients underwent 491 total procedures. Eighty-three percent of reported injection indications were for febrile illness. Twenty-five percent of reported injections explicitly identified quinine as the agent. Although ten different procedures were performed, achilles tendon lengthening, triple arthrodesis, tibialis posterior and anterior tendon transfers composed 83% of all conducted surgeries. Amongst five different foot deformities, equinus and varus were most likely to undergo soft tissue and bony procedures, respectively (*p*=0.0223). Ninteen percent of patients received two or more surgeries. Sixty-seven percent of patients achieved a plantigrade outcome; 13.61% had not by the end of the study period; 19.3% had unreported outcomes. Those who lived further from the facility had longer times between the inciting injection and initial hospital presentation (*p*=0.0216) and were more likely to be lost to follow-up (*p*=0.0042).

**Conclusion:**

PIP is a serious iatrogenic disability. Prevention strategies are imperative, as over 400 children required 491 total surgical procedures within just six years at one hospital in Uganda.

**Supplementary Information:**

The online version contains supplementary material available at 10.1186/s12891-022-05664-4.

## Introduction

Post-injection paralysis (PIP) is paralysis that occurs distally in a limb immediately after an injection is given proximally in the same limb, without any other possible cause for the paralysis [1, 2]. The paralysis may present as early as 30 minutes to over seven days, with 88% of patients having immediate onset [3, 4]. The World Health Organization has estimated that six billion injections are unsafely administered, and nine billion injections are unnecessarily administered globally each year [5]. In Uganda, iatrogenic injection injury is a major cause of disability in children [6]. Injections are commonly given by trained and untrained providers in hospitals, health centers, private clinics, and homes, with 63 to 83% of Ugandan households owning needles and syringes [6].

**Fig. 1:**
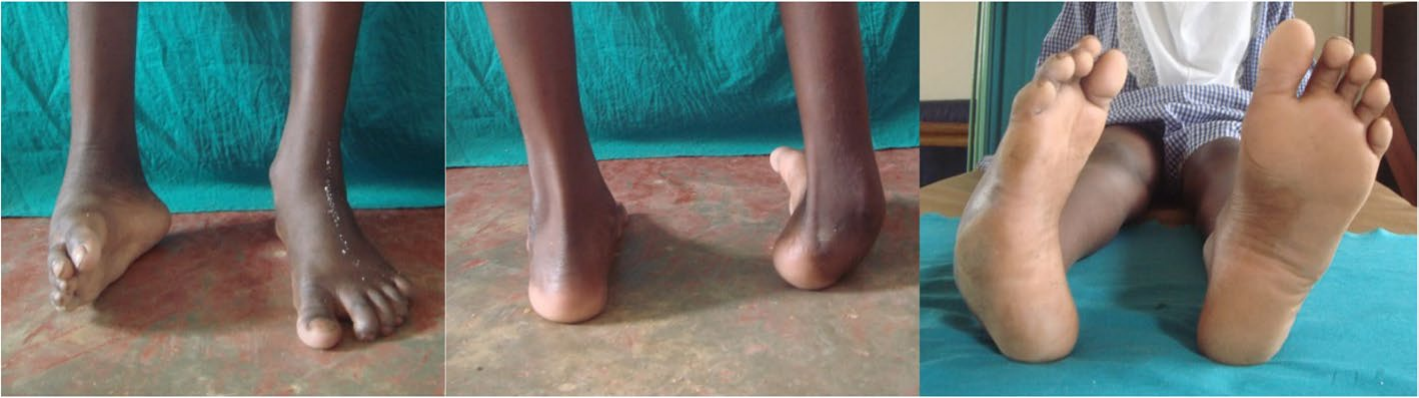
Clinical images of a nine-year-old female who presented to us after having sustained an injection injury five years prior (substance not known by family), following a short febrile illness. She presented with this semi-rigid foot deformity of cavus, adductus, varus and equinus, which developed over time after the injection. Sensation in the foot was reported to be normal and there was significant motor weakness in the peroneal tendons

Sciatic nerve injury that does not self-resolve, with or without supportive treatment, often results in a chronic foot deformity. Severity of the deformity is determined by the extent of the nerve injury and which distal muscles become dysfunctional [7]. Sciatic nerve injury may arise when the gluteal injection bathes the nerve with a neurotoxic substance, like quinine [6, 8], or when the injection is not administered in the correct location and the nerve is directly traumatized by contact or pressure/compression. An injection directly into the sciatic nerve causes the most neuronal destruction, resulting in almost immediate onset of pain, paresthesia, and motor deficit [3], which are thought to arise from direct damage to axons and Schwann cells, with further breakdown of the blood-nerve barrier by the injected neurotoxic chemical [4]. Resultant deformities seen in PIP can significantly range from simple equinus to a true equinocavovarus foot, much like a clubfoot [9]. A clinical example of a PIP foot deformity is shown in Figure 1.

There is scarce orthopaedic literature that addresses PIP, particularly the epidemiology, outcomes, and efficacy of current surgical interventions and the impact this iatrogenic condition has on the lives of children. Affected individuals and their communities may lack the awareness of available treatment options and the understanding that delayed intervention may result in greater disability and difficulty when treating the foot deformity.

To expand the current literature addressing PIP, this study investigates the epidemiology and treatment for PIP patients treated at a single hospital in Uganda - the CoRSU Rehabilitation Hospital in Kisubi.

## Materials and Methods

### Design

This is a retrospective case series of all pediatric patients (<18 years old) surgically treated for PIP at the CoRSU Rehabilitation Hospital in Kisubi, Uganda from 1st January 2013 to 31st December 2018.

### Setting

The CoRSU Rehabilitation Hospital is a private, non-profit, non-governmental organization funded hospital located in Kisubi, Uganda - just outside the capital city of Kampala. CoRSU is a Ugandan initiative encouraged and supported by international and local donors. Patients below 17 years of age receive free and subsidized treatments, including surgery and rehabilitation [10].

This study was approved by the Mildmay Uganda Research Ethics Committee, the CoRSU Hospital Research Committee, the Uganda National Council for Science and Technology, and the University of California San Francisco (UCSF) Institutional Review Board.

### Sample

The hospital records were queried for any patient that underwent surgery for PIP between 1st January 2013 and 31st December 2018. Inclusion criteria included patients less than 18 years old who received surgical treatment for the diagnosis of PIP at the CoRSU Rehabilitation Hospital. This study excluded patients with PIP who were seen in the outpatient clinic and did not have a surgery performed at the CoRSU Rehabilitation Hospital or who had foot deformities for some reason other than PIP, including polio. Of note, the last confirmed case of polio in Uganda was in 1996 [11], and all but five patients in our cohort were born in 1997 or later. For those five born in 1996, the medical records had no mention of polio history. Given the rarity of polio at that time and prevalence of PIP in Uganda, the assumption is that these five patients had PIP. There was no specific documentation of maintenance of extensor hallucis longus function and/or sensation which would be seen in polio.

### Data sources and data elements:

Patient information was obtained from written patient charts and the electronic health record system at the CoRSU Rehabilitation Hospital. Information was collected into a password-protected Excel database on an encrypted computer.

The following data were collected from patient charts: age at presentation, age at initial surgery, sex, weight, indication for injection, geographic district, pre-surgical foot deformity, side of procedure, pre-treatment complications, type of conducted surgical procedure, use of brace, post-operative physical therapy follow-up, number of surgeries per patient, post-operative complications, and whether a plantigrade outcome was achieved. The treatment algorithm was not explicitly recorded in the patient charts. The dataset of the raw data used in this study is provided in Supplementary Table 1.

### Data management

Coding was performed for multiple variables during the data analysis. Codes for pre-operative foot deformities included: equinus, equinovarus (including equinocavovarus and equinocavus), varus, cavovarus, calcaneus (including calcaneovarus and calcaneovalgus), and unknown for those without a pre-operative deformity recorded in the chart. Foot deformity codes were based off of previous descriptions of foot deformities [9]. Codes for injection indication include commonly reported indications for unregulated injections in the literature [8, 12, 13]: malaria treatment, febrile illness, immunization, and other. Codes for pre-operative complications included: ulcer, skin callus, superficial wound. Codes for types of surgical procedures conducted included: achilles tendon lengthening, triple arthrodesis, tibialis anterior tendon transfer, tibialis posterior tendon transfer, calcaneal osteotomy, plantar fasciotomy, manipulation and casting, mid tarsal osteotomy, shortening of the lateral column, and other. Depending on the combination of procedures performed, cases were further categorized into four surgical groups of soft tissue procedures, bony procedures, combined, and manipulation and casting (no surgery).

Post-operative complications included: pressure related issues (i.e., pressure ulcer, pressure sore, blister, persistent skin callus), surgical site issue (i.e., stitch abscess, granuloma, surgical site infection, wound dehiscence), and non-surgical site infection (i.e., parasitic infection, non-surgical site infection). Post-operative outcomes were plantigrade outcome, no plantigrade outcome but with planned surgeries, no plantigrade outcome without documented planned surgeries, and unreported plantigrade outcome or lost to follow-up. Accuracy of coding was verified by the Principal Investigator.

### Statistical analysis

Descriptive statistics were utilized to report frequency and percentages of demographic and pre-operative and post-operative factors. Distance from patients’ home districts to CoRSU Hospital was calculated using Google Maps. For the predictor variables involving age, patients were grouped into four age categories: 0-4, 5-9, 10-14, and 15+ years, which follow clinical bone maturation stages. Distance from hometown to CoRSU was grouped into four categories: 0-40, 41-100, 101-250, and 251+ km. Variables involving age, distance, and time were separated into four equal categories, as well as calculated as continuous variables. Chi square tests, analyses of variance, and linear regression models were conducted to determine any statistically significant associations between two categorical, categorical and continuous, and two continuous variables, respectively. Statistical significance was defined as p-value <0.05.

## Results

Within the six-year period, 402 patients were surgically treated for PIP at the CoRSU Rehabilitation Hospital in Kisubi, Uganda. There was a 1.3:1 male to female distribution of patients (Table 1). Forty-seven percent of patient charts explicitly documented the date of the inciting injection, ranging from 0.04 to 15.11 years (3.49 ± 2.87). Age at presentation ranged from 0.57 to 16.10 years (9.12 ± 3.94). Time from date of injection to age at presentation varied from 0.12 to 15.85 years (5.37 ± 3.50). Fourteen patients were given their inciting injection for malaria treatment, 37 for unspecified febrile illness (which is often presumed as malaria in this endemic area), and nine for other reasons [2]. Of the 174 cases that reported receiving an IM injection, 25% explicitly mentioned quinine as the main injection agent. 58.31%, 41.19%, and <1% of patients had right foot, left foot, and bilateral foot deformities, respectively. Thirty patients presented with skin complications including ulcer (16), skin callus (11), and superficial wound (3). Patients were coded into six different foot deformity categories of equinus (99), equinovarus (188), varus (22), cavovarus (23), calcaneus (3), and unknown (67).

**Table 1:** Demographics and clinical features of pediatric cases surgically treated for PIP. The numbers of age and time are averages with respective standard deviations

Variable	Number	% of total patients with reported data (*n*=402)
Total number of patients	402	100
Sex		
Male	228	56.72
Female	173	43.03
Unspecified	1	0.25
Patients with known date of injection (%)	188	46.77
Avg age when received inciting injection (yrs)	3.49 ± 2.87	
# of pts 0 – 4 yrs	137	34.08
# of pts 5 – 9 yrs	40	10.00
# of pts 10 – 17 yrs	11	2.74
Avg age at initial presentation to CoRSU hospital (yrs)	9.12 ± 3.94	
# of pts 0 – 4 yrs	55	13.68
# of pts 5 – 9 yrs	169	42.04
# of pts 10 – 14 yrs	132	32.84
# of pts 15 – 17 yrs	46	11.44
Time from date of injection to initial presentation (yrs)	5.37 ± 3.50	
Distance from home district to CoRSU (km)	154.66 ± 159.78	
# of pts 0 – 40 km	137	34.08
# of pts 41 – 100 km	66	16.41
# of pts 101 – 250 km	98	24.38
# of pts 251+ km	94	23.34
Reported indications for injection	60	14.93
Unspecified febrile illness	37	9.20
Malaria	14	3.48
Immunization	4	1.00
Other	5	1.24
Affected PIP-side		
Right	234	58.21
Left	166	41.29
Bilateral	2	0.50
Patients with pre-treatment	30	7.46
skin complications Skin callus	11	2.74
Superficial wound	3	0.75
Ulcer	16	3.98
Foot deformity characterization		
Equinus	99	24.63
Equinovarus	188	46.77
Varus	22	5.47
Cavovarus	23	5.72
Calcaneus	3	0.75
Unknown	67	16.67

**Fig. 2:**
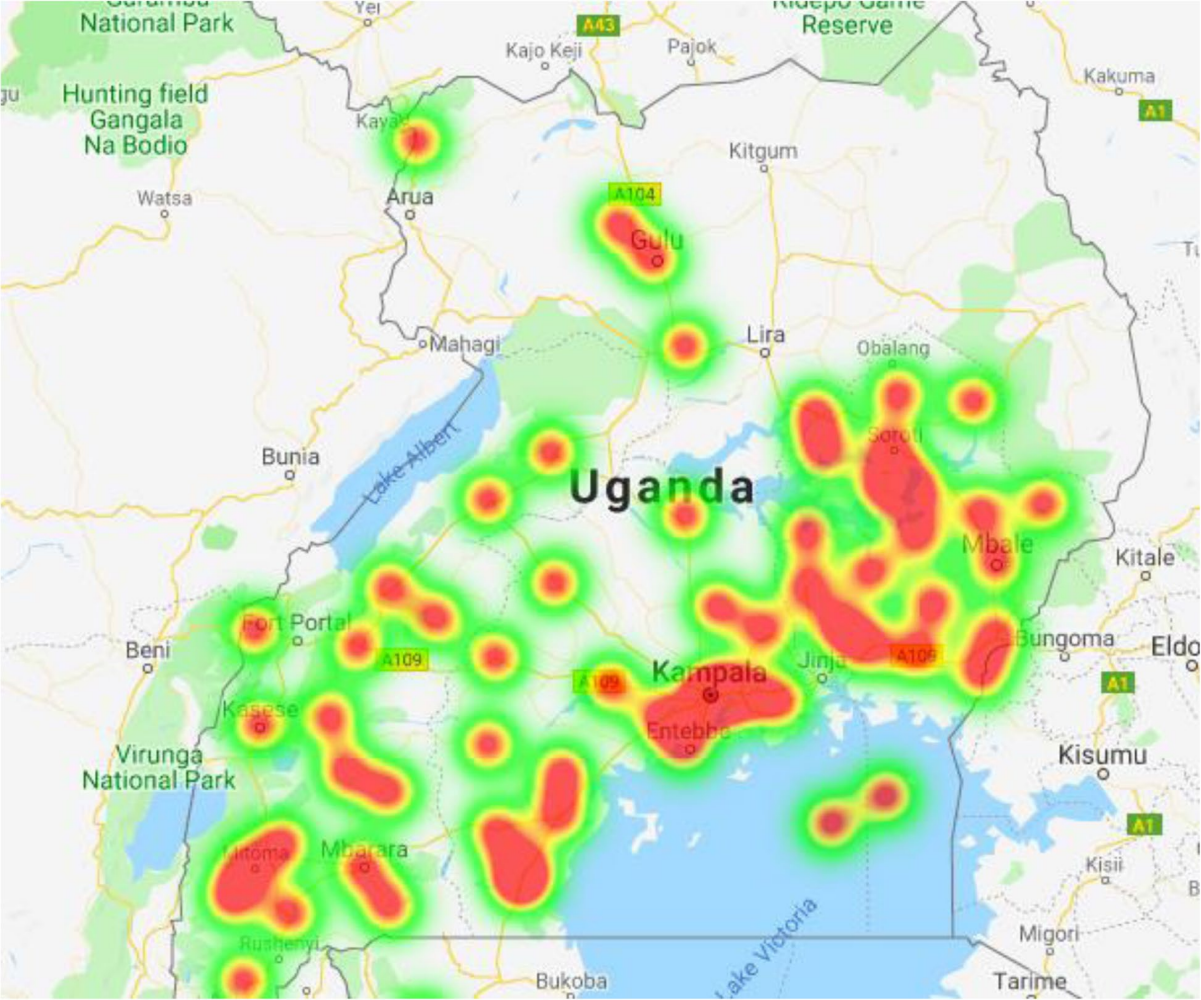
Heat map depicting the wide distribution of the study patients’ home districts and the relative volume contribution from those areas. Red, yellow, and green shading represent areas with the greatest, fewer, and fewest number of cases. This figure does not show the one case from South Sudan and the three cases from the Democratic Republic of Congo

Figure 2 depicts a heat map of the home districts the patients came from. There is widespread distribution of patients geographically from throughout Uganda. The distance from home district to CoRSU ranged from 6.5 to 1318 km (154.66 ± 159.78) (Table 1). Around thirty-four percent of patients lived within 40 km of the Hospital (Table 1). All patients were from Uganda, except for one from South Sudan and three from the Democratic Republic of Congo.

**Fig. 3:**
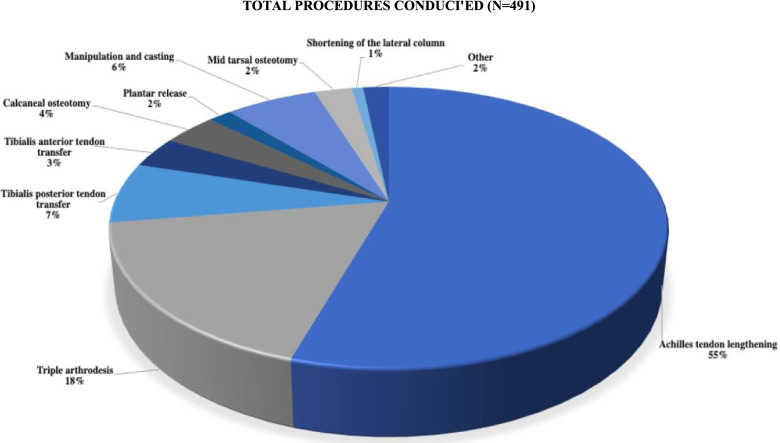
Total procedures conducted from 1st January 2013 to 31st December 2018 for PIP - Achilles tendon lengthening (55%), triple arthrodesis (18%), tibialis posterior tendon transfer (7%), manipulation and casting (6%), calcaneal osteotomy (4%), tibialis anterior tendon transfer (3%), mid tarsal osteotomy (2%), plantar release (2%), shortening of the lateral column (1%), and other (2%), with respective percentages of the total procedures conducted

Within this population of 402 children, 491 total procedures were performed. (Fig. 3). The age at first surgery ranged from six months to 16.10 years (9.43 ± 3.94) (Table 2). The most commonly performed surgical procedures were Achilles tendon lengthening (55%), triple arthrodesis (18%), tibialis posterior tendon transfer (7%), and manipulation and casting (6%) (Fig 3). Patients underwent varied numbers of operations (ranging from one to four) and had a unique combination of performed surgical procedures.

**Table 2:** Perioperative characteristics and outcomes of surgically treated pediatric PIP cases. Percentages of total patient cases are provided

Variable	No. of cases	% (*n*=402)
Total surgical procedures conducted	491	
Average age at first surgery	9.44 ± 3.94	
# of pts 0 – 4 yrs	39	9.70
# of pts 5 – 9 yrs	174	43.28
# of pts 10 – 14 yrs	135	33.58
# of pts 15 – 17 yrs	54	13.43
Number of patients who received		
Only one surgical procedure	326	81.09
Two surgical procedures	63	15.67
Three or more surgical procedures	13	3.23
Average number of surgeries per patient	1.25 ± 0.50	
Received physical therapy	213	52.98
Used AFO brace	297	73.88
Number of patients with post-treatment complications	33	8.21
Pressure-related complication	20	5.00
Surgical site complication	9	2.23
Non-surgical site infection	4	1.00
Plantigrade foot		
Yes	270	67.16
No, with future planned surgery	25	6.21
No, without future planned surgery	30	7.46
Unreported/ lost to follow-up	77	19.15

Based on their unique procedural combinations, patients were further grouped into one of four different categories by the procedures they had received. Two hundred and seventy-seven patients underwent soft tissue-only procedures, 74 patients underwent only bony procedures, 40 patients underwent combined (soft-tissue and bone) procedures, and 11 patients received manipulation and casting procedures (non-surgical). Three hundred and twenty-six (81.09%) patients received one surgical procedure, 63 (15.67%) underwent two surgical procedures, and 13 (3.23%) received three or more surgical procedures, with an average of 1.25 surgical procedures per patient (Table 2).

Table 2 shows various perioperative characteristics and outcomes. Physical therapy was documented in 213 (52.98%) patient charts. Two hundred and ninety-seven (73.88%) patients were prescribed an AFO brace. Thirty-three patient charts mentioned a post-treatment complication, with 20 patients reporting a pressure-related complication (i.e., pressure ulcer, pressure sore, blister, or persistent skin callus), nine patients with a surgical site problem (i.e., stitch abscess, granuloma, surgical site infection, wound dehiscence), and four patients with a non-surgical site infection (i.e., parasitic infection not related to surgery or the surgical site, non-surgical site infection). Two hundred and seventy (67.16%) patients reported plantigrade foot, 25 (6.21%) cases did not have plantigrade foot but had planned future surgeries, 30 (7.46%) patients did not have plantigrade foot and no additional treatment documented, and 77 (19.15%) cases had no recorded information regarding post-operative foot position. Many of the non-plantigrade patients were expected to follow-up in clinic or for surgery but did not.

**Fig. 4:**
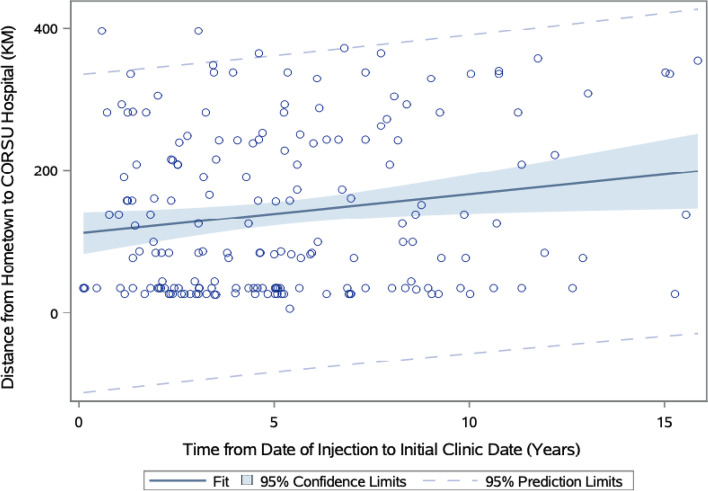
Linear regression model of distance from hometown to CoRSU predicting time between date of inciting injection to age at presentation. Further distances from CoRSU predict longer times between date of injection to age at presentation (*p*=.0216)

**Fig. 5:**
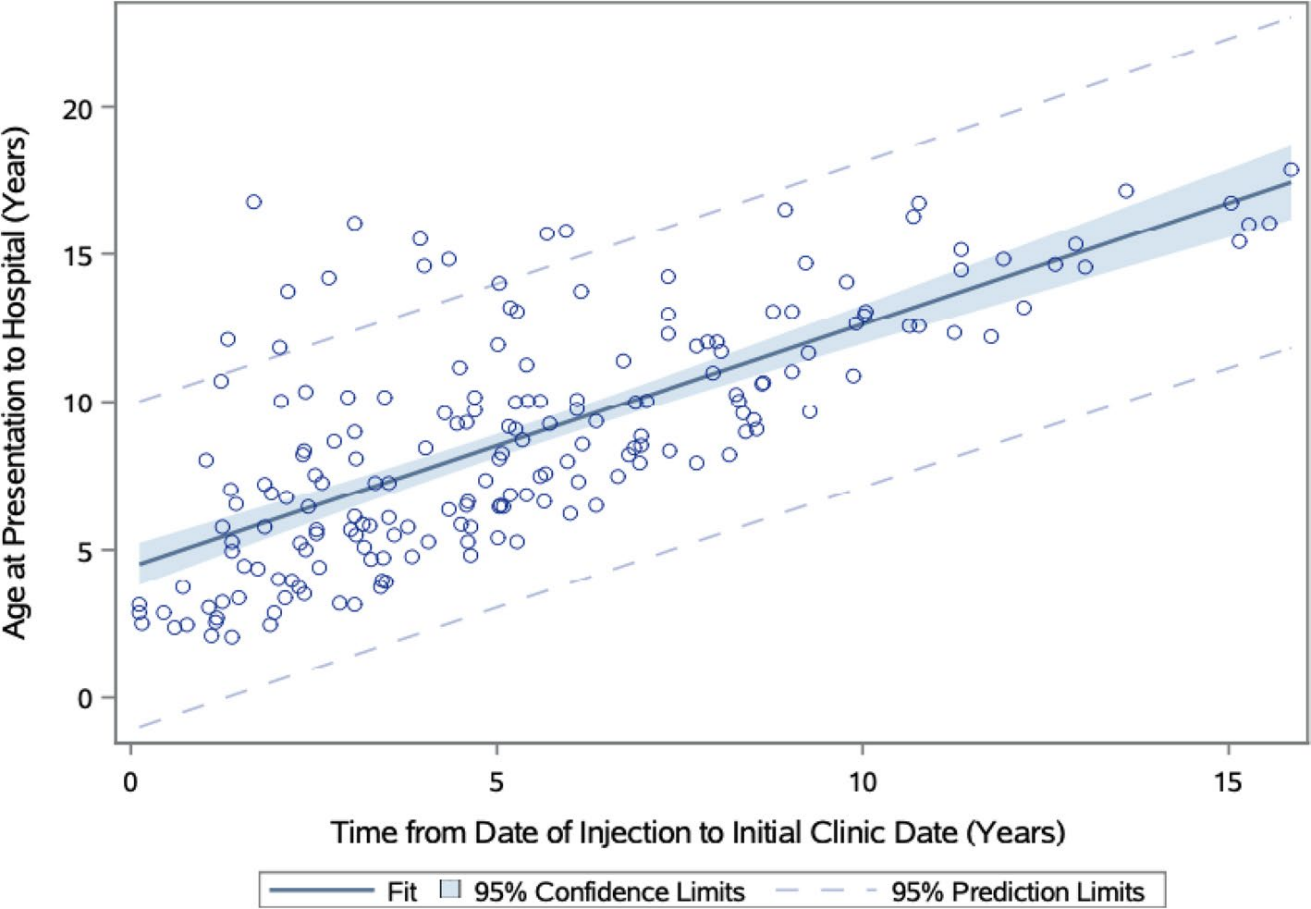
Linear regression model of age at presentation predicting time from date of inciting injection to age at presentation Older ages at initial presentation predict longer times between date of inciting injection to initial age at presentation (*p*<0.001)

Statistically significant associations using chi-square tests between categorical variables are reported in Table 3 (*p*<0.05). Appropriately, the pre-surgical foot deformity determined which combination of surgical procedures were performed, with equinus, varus, and calcaneus foot deformities undergoing the most soft tissue, bony, and combined surgical procedures, respectively (*p*=0.0223). Those who were lost to follow-up were more likely to live farther away from CoRSU (*p*=0.0042). As depicted by Figs. 4 and 5, farther distances from CoRSU and older ages at presentation were more likely to predict longer times between receiving the inciting injection to age at presentation (*p*=0.0216 and *p*=<0.001, respectively).

**Table 3:** Correlations between pre-operative factors and post-operative outcomes.

Predictor variable	Outcome variable (# of pts)				*p*-value
Lost to follow-up	Distance from hometown to CoRSU (km)				*0.0042*
	0-40	41-100	101-250	251+	
No	124	53	77	67	
Yes	13	13	21	27	
Pre-surgical foot deformity	Categorization of performed surgical procedures				*0.0223*
	Soft tissue	Bony	Combined	M&C^a^	
Equinus	80	6	12	2	
Equinovarus (including equinocavovarus)	125	37	21	5	
Varus	13	8	1	0	
Cavovarus	12	8	3	0	
Calcaneus	2	0	1	0	

Chi-square tests were calculated for categorical associations. Statistical significance of *p*<0.05

^a^manipulation and casting

## Discussion

Post-injection paralysis is a serious, preventable iatrogenic disability. Within a six-year time period, over 400 children were surgically treated for PIP-induced foot deformities at this one hospital in Uganda. Seventy-six patients required multiple surgeries during the study time period, with another 25 identified as needing additional surgery in the future. Over 490 surgical procedures were performed, emphasizing the immense time and resources allocated to manage this preventable disability.

To the best of our knowledge, this study presents the largest evaluation of epidemiological factors and surgical intervention of post-injection paralysis. This study reports that 80% of injection indications were due to febrile illness, supporting prior observations that intramuscular injections are usually recommended for children with febrile illnesses, including malaria [12]. The lack of injection regulation and easy accessibility to injections may further explain the high prevalence of PIP in certain parts of Uganda [13]. Although only 44 cases explicitly reported using quinine injections, we surmise that quinine may play a significant role in the pathogenesis of PIP as reported by Alves et al. [4, 5, 8]

In lower-income regions, the commute to healthcare facilities may pose a substantial barrier to care, as evidenced by greater loss of follow-up in patients who lived farther away from CoRSU. Farther distances from the hospital also predicted longer times between onset of paralysis to getting surgical care. The statistically significant association between pre-surgical foot deformity and type of surgical procedure performed supports both surgical logic and prior published treatment recommendations [9].

There were several limitations of this study. The most significant limitation is the study’s retrospective nature with a lack of standardized data charting by surgeons. Therefore, the pre-treatment foot deformity, specific motor and sensory findings on physical examination of the leg, outcome, and complications were not necessarily reported in a standard fashion, or at all, in the records. Given limited data provided in these patient records regarding muscle strength and function, we cannot evaluate the association between pre-operative function and surgical procedure performed. The role of serial casting pre-operatively also warrants exploration, but there is not sufficient data in our current cohort to assess the role of casting to improve the deformity and perhaps decrease the complexity of surgery needed and/or improve outcomes.

Injection dates, substances injected, and personnel who administered the injection were self-reported from parental recollection and are subject to recall bias. Birthdates for children in Uganda are sometimes not exactly known, so were estimated for some children in their medical charts. Nutritional status could not be assessed as the majority of weights and mid-upper arm circumference were not reported. Patients also varied in follow-up for physical therapy in terms of number of sessions and timing in relation to surgery. We also do not have patient reported outcome data on these patients, including an understanding of their pain, the impact of the condition, subsequent treatment, and their quality of life.

Furthermore, evaluations of the timing of nerve injury, denervation type, and outcome of related muscles are important to consider when discussing prognosis and management of PIP. However, the limited chart data precludes substantial investigation of such factors. Further research, including a well-designed prospective study, is necessary to study and characterize the spectrum of motor and sensory dysfunction that can result from PIP in order to improve current treatment algorithms.

Despite these limitations, this study still reports on the largest cohort of patients with PIP described in the literature. These findings emphasize the serious risk of gluteal intramuscular injections. With the average time of 5.37 years between the date of injection to age at presentation, a majority of these vulnerable patients presented to surgical care with a long-standing deformity. This extended period of time may partly explain the high volume of cases without reported outcome of a plantigrade foot as the foot deformity may become quite stiff with secondary bone changes and limit surgical options and successful outcomes.

## Conclusions

PIP is a disabling orthopaedic condition which is preventable. These cases are widely distributed throughout Uganda, indicating the widespread injection practices that put the sciatic nerve at risk. Therefore, greater effort is warranted to prevent this problem, since it is difficult to manage and achieve a successful outcome once the injury is present. The high volume of surgeries required for these iatrogenically-injured patients utilizes scarce hospital and surgical resources that could otherwise be allocated for non-preventable conditions and unavoidable traumatic injuries in this pediatric population. Future research should evaluate potential preventative interventions, including reevaluating current malaria testing and treatment protocols, implementing greater regulation of injection distribution at pharmacies, providing more education to healthcare staff on safe injection practices, and preventing untrained persons from administering injections.

## Supplementary Information

**Additional file 1.**

## Data Availability

Electronic and patient health records were accessed at the CoRSU Rehabilitation Hospital in Kisubi, Uganda. The datasets used and/or analyzed during the current study are available from the corresponding author on reasonable request.

## Abbreviations

PIP: Post-injection paralysis
